# Differential Genomic Profile in *TERT, DSP*, and *FAM13A* Between COPD Patients With Emphysema, IPF, and CPFE Syndrome

**DOI:** 10.3389/fmed.2021.725144

**Published:** 2021-08-19

**Authors:** Javier Guzmán-Vargas, Enrique Ambrocio-Ortiz, Gloria Pérez-Rubio, Marco Antonio Ponce-Gallegos, Rafael de Jesus Hernández-Zenteno, Mayra Mejía, Alejandra Ramírez-Venegas, Ivette Buendia-Roldan, Ramcés Falfán-Valencia

**Affiliations:** ^1^HLA Laboratory, Instituto Nacional de Enfermedades Respiratorias Ismael Cosío Villegas, Mexico City, Mexico; ^2^COPD Clinic, Instituto Nacional de Enfermedades Respiratorias Ismael Cosío Villegas, Mexico City, Mexico; ^3^Interstitial Pulmonary Diseases and Rheumatology Unit, Instituto Nacional de Enfermedades Respiratorias Ismael Cosío Villegas, Mexico City, Mexico; ^4^Tobacco Smoking and COPD Research Department, Instituto Nacional de Enfermedades Respiratorias Ismael Cosío Villegas, Mexico City, Mexico; ^5^Translational Research Laboratory on Aging and Pulmonary Fibrosis, Instituto Nacional de Enfermedades Respiratorias Ismael Cosío Villegas, Mexico City, Mexico

**Keywords:** emphysema, idiopathic pulmonary fibrosis, CPFE, COPD, SNP

## Abstract

**Background:** Genetic association studies have identified single nucleotide polymorphisms (SNPs) associated with lasting lung diseases such as Chronic Obstructive Pulmonary Disease (COPD) and Idiopathic Pulmonary Fibrosis (IPF), as well as the simultaneous presentation, known as Combined Pulmonary Fibrosis and Emphysema (CPFE) Syndrome. It is unknown if these diseases share genetic variants previously described in an independent way. This study aims to identify common or differential variants between COPD, IPF, and CPFE.

**Materials and methods:** The association analysis was carried out through a case-control design in a Mexican mestizo population (*n* = 828); three patients' groups were included: COPD smokers (COPD-S, *n* = 178), IPF patients (*n* = 93), and CPFE patients (*n* = 16). Also, two comparison groups were analyzed: smokers without COPD (SWOC, *n* = 367) and healthy subjects belonging to the Mexican Pulmonary Aging Cohort (PAC, *n* = 174). Five SNPs in four genes previously associated to interstitial and obstructive diseases were selected: rs2609255 (*FAM13A*), rs2736100 (*TERT*), rs2076295 (*DSP*) rs5743890, and rs111521887 (*TOLLIP*). Genotyping was performed by qPCR using predesigned Taqman probes.

**Results:** In comparing IPF vs. PAC, significant differences were found in the frequency of the rs260955 G allele associated with the IPF risk (OR = 1.68, *p* = 0.01). Also, the genotypes, GG of rs260955 (OR = 2.86, *p* = 0.01) and TT of rs2076295 (OR = 1.79, *p* = 0.03) were associated with an increased risk of IPF; after adjusting by covariables, only the rs260955 G allele remain significant (*p* = 0.01). For the CPFE vs. PAC comparison, an increased CPFE risk was identified since there is a difference in the rs2736100 C allele (OR = 4.02, *p* < 0.01; adjusted *p* < 0.01). For COPD-S, the rs2609255 TG genotype was associated with increased COPD risk after adjusting by covariables.

**Conclusion:** The rs2736100 C allele is associated with decreased IPF risk and confers an increased risk for CPFE. Also, the rs2076295 TT genotype is associated with increased IPF risk, while the GG genotype is associated with CFPE susceptibility. The rs2609255 G allele and GG genotype are associated with IPF susceptibility, while the TG genotype is present in patients with emphysema.

## Introduction

Chronic Obstructive Pulmonary Disease (COPD) and Idiopathic Pulmonary Fibrosis (IPF) are the leading causes of morbidity and mortality of pulmonary etiology in individuals over the fifth decade of life (1, 2); each one with an independent pathophysiological and clinical behavior. In the last 30 years, a new entity where both diseases can coexist has been described, and this is the Combined Pulmonary Fibrosis and Emphysema (CPFE) syndrome with a worse prognosis and higher mortality compared with patients with individual diseases (3–5).

One of COPD's main characteristics is emphysema, defined as the irreversible destruction of the alveolar wall beyond the terminal bronchiole; however, not all COPD patients present it (3, 5–7). On the other hand, IPF is another lung disease with a fatal prognosis and survival of ~5 years from diagnosis (8, 9). It results from the combination of excessive extracellular matrix production, loss of the alveolar epithelium, and permanent collapse of the alveolar sacs (5).

COPD is a multifactorial disease, and tobacco smoking is the main risk factor described; however, only around 20% of smokers will develop COPD, suggesting that other factors such as genetics can influence the disease susceptibility. The most representative is the genetic α1 antitrypsin deficiency, but its frequency is low and mainly in the European population. Nevertheless, genome-wide association studies have identified genetic markers, mainly SNPs, in susceptibility to COPD; for example, at least 20 polymorphisms in matrix metalloproteinases (MMP) genes have been associated with this disease. The *MPP1* rs1799750 has been associated with an apical distribution of emphysema in these patients (10, 11).

The CPFE is a recently described entity in which emphysema is predominantly in the upper and fibrosis in the basal lung lobes and subpleural regions (9, 12). The etiology and pathogenesis of this disease remain unknown and result in a diagnostic challenge with poor prognosis and high mortality (4, 12, 13). In 2020, Kinjo et al. reported that the minor allele of the *AGER* rs2070600 was associated with CPFE related with COPD patients in the dominant model in a Japanese cohort (14). Variants in MMP are also described in IPF and CPPE; for instance, Xu et al. reported that the T allele of *MMP9* (C-1562T) might predispose to the development of emphysema in patients with IPF in a Chinese population (15). If an interaction between COPD and IPF can result in the development of CPFE remains unclear since each one has a different pathophysiological profile. However, both entities share genetic variants that had been independently associated. Among the most reported are single-nucleotide variants in *MUC5B, FAM13A, DSP*, and *TERT* genes (16, 17).

We hypothesize that SNP-type variants are involved in the susceptibility and pathogenesis of CPFE and present a differential profile in COPD with emphysema and IPF. This study aims to identify differential variants between COPD, IPF, and CPFE in patients from a mestizo-Mexican population.

## Methods

### Study Population

In this case-control study, a total of 828 participants divided into three groups of patients were included; COPD smokers (COPD-S), Idiopathic Pulmonary Fibrosis (IPF) patients, and subjects with Combined Pulmonary Fibrosis and Emphysema (CPFE) syndrome diagnosis; as a control groups, smokers without COPD (SWOC), and healthy subjects belonging to the Pulmonary Aging Cohort (PAC) (18, 19) over 50 years were included.

The diagnosis of COPD was confirmed through spirometry, from the FEV_1_/FVC ratio <70% after the bronchodilator administration, taking as reference the values for Mexicans defined by Pérez-Padilla et al. (20).

The control groups included smokers without COPD with normal spirometry values (FEV_1_/FVC >70%). Individuals with a tobacco index (TI) >10 packs/year and indistinct gender were included; patients with clinical evidence of other bronchopulmonary diseases were excluded. We also include subjects with the presence of emphysema with at least 10% of extension in tomography (9). Participants were recruited from 2009 to 2016.

The IPF diagnosis was established considering the criteria of the 2018 ATS/ERS/JRS/ALAT guidelines (tomographic or histopathological pattern of usual interstitial pneumonia) (21). Smokers, and non-smokers, of both sexes, were included. Patients with evidence of a secondary cause of fibrosis (autoimmune processes, systemic diseases, or a history of exposure) were excluded. Recruitment was carried out in the period 2013–2019. The control group from the pulmonary aging cohort was randomly selected (18), matching the variables of age, sex ratio, and smoking history of the IPF group. All subjects are defined as pulmonary healthy by spirometry (using the reference values of Pérez-Padilla) or imaging (20).

The CPFE diagnosis was determined through high-resolution tomography, where there were upper emphysematous lesions and fibrosis in the lower lobes in the subpleural region. Smokers subjects without evidence of other pulmonary or systemic disease were included. Recruitment began in 2019 but was suspended due to the COVID-19 lockdown. For this patients' group, the subjects of the PAC were considered as controls.

Participants in the COPD-S, SWOC, and CPFE groups were recruited from the Tobacco Smoking and COPD Research Department, and the clinical service 5. In addition, the IPF group was evaluated and managed in the Interstitial Lung Disease and Rheumatology Unit (ILD&RU), while the PAC participants from the Translational Research Laboratory on Aging and Pulmonary Fibrosis of the “Moises Selman Lama” Research Unit. All the departments above are part of the Instituto Nacional de Enfermedades Respiratorias Ismael Cosio Villegas (INER) at Mexico City, Mexico.

Clinical and demographic selection of case and control groups are shown in Supplementary Figure 1.

### Obtaining and Processing Biological Samples

DNA was extracted from peripheral blood mononuclear cells (PBMC) obtained through venipuncture in EDTA tubes. The genetic material was extracted with the commercial BDtract Genomic DNA isolation kit (Maxim Biotech, San Francisco, CA, USA) and rehydrated in TE buffer. The DNA was quantified through UV absorption spectrophotometry at a wavelength of 260 nm using the Nanodrop 2000 equipment (Thermo Scientific, Wilmington, DE, USA).

### Selection of SNPs

The SNPs were selected based on a bibliographic search in PubMed (NCBI), identifying polymorphisms previously associated with IPF, emphysema, and CPFE. We also considered those SNPs that had a minor allele frequency (MAF) > 5%. Five SNPs were evaluated: rs2609255 (*FAM13A*), rs2736100 (*TERT*), rs2076295 (*DSP*), rs5743890 and rs111521887 (*TOLLIP*), all these are intronic variants. Supplementary Table 1 shows SNPs' molecular characteristics.

### Genotyping

Allele discrimination of SNPs was carried out using Taqman probe technology with predesigned assays for each polymorphism: rs2609255 (C__15906608_10), rs2736100 (C___1844009_10), rs2076295 (C__16167921_10), rs5743890 (C__15906608_10), rs2736100 (C___1844009_10), rs2076295 (C__16167921_10), rs5743890 (C__15902) (San Francisco California, USA) at a concentration of 20X. Genotyping was performed using qPCR on the 7300 Real-Time PCR System (Applied Biosystems, San Francisco, CA, USA). Allele discrimination was performed by the application SDS (sequence detection software) v. 1.4 (Applied Biosystems, San Francisco, CA, USA).

### Statistical Analysis

The analysis and comparison of the clinical and demographic variables of the comparison groups were performed using the RStudio software (22). The normality of the variables was evaluated through the Kolmogorov-Smirnov normality test; thus, it was determined to use non-parametric statistics. The comparison of quantitative variables between groups was carried out using the Mann–Whitney *U*-test, and the frequency of qualitative variables was compared with the χ^2^-test.

Allele frequency and genotype analysis were performed using Epi Info 7.1.4.0 software (23) (Centers for Disease Control and Prevention, Atlanta, GA, USA) using the χ^2^-test and Fisher's exact test (when the frequency of a variable was <10) to obtain the 95% confidence intervals and the OR values. The Hardy–Weinberg equilibrium of the variants was calculated using the PLINK v1.07 software (24). The results and associations obtained were considered significant when a *p* < 0.05. Logistic regression analysis was performed to adjust for possible confounding variables using PLINK v. 1.07.

The univariate and multivariate logistic regression model was designed to evaluate the relationship of the genotypes of the polymorphisms with clinical variables related to prognosis and development of the disease in RStudio.

### Ethical Approval and Informed Consent

This study was reviewed and accepted by the Institutional Committees for Research, Ethics in Research, and Biosecurity of the INER (approval numbers: C09-19 and C39-14). In addition, all participants signed the written informed consent form and provided a privacy statement that describes the legal protection of their data, both documents approved by the Institutional Research and, Ethics in Research Committees.

All experiments were performed following pertinent regulations and considering the STREGA (STrengthening the REporting of Genetic Association) guidelines to design this genetic association study.

## Results

### Demographic Variables in Cases and Controls

Three case-control comparisons were included; the first refers to COPD-smokers patients (178 COPD-S) and smokers without COPD (367 SWOC). The second includes 93 IPF patients vs. 174 PAC subjects, and the third comparison consists of 16 CPFE vs. 174 PAC. The clinical and demographic variables are shown in Table 1. When comparing COPD-S vs. SWOC, significant differences were found in age (*p* < 0.01), male sex (*p* < 0.01), being found more frequently in COPD-S. Also, the BMI was lower in the COPD-S group than the SWOC group, and TI was higher in the COPD-S group (*p* < 0.01). In the IPF vs. PAC comparison, no differences were found between the demographic variables. However, when comparing CPFE vs. PAC, statistically significant differences were found in TI (*p* < 0.01).

**Table 1:** Comparison of demographic variables between COPD patients vs. SWOC subjects; IPF patients vs. PAC subjects; CPFE patients vs. PAC subjects.

	**COPD-S (*n* = 178)**	**SWOC (*n* = 367)**	***p***	**IPF (*n* = 93)**	**CPFE (*n* = 16)**	**PAC (*n* = 174)**	***p*** ^*^	***p*** ^**^
Age (years)	69 (62–74)	63 (58–70)	<0.01	67 (60–72)	70 (68–74)	67 (63–73)	0.22	0.18
Sex % (M/F)	77.17/22.83	46.46/53.54	0.01	75.26/24.74	68.75/31.25	67.81/32.19	0.26	0.92
BMI (kg/m^2^)	25.16 (21.79–29.41)	27.01 (24.17–30.25)	<0.01	26.60 (23.48–30.10)	27.5 (22.96–28.41)	27.67 (25.02–30.43)	0.07	0.29
TI (packs/year)	43.00 (28.42–65.62)	27.00 (17.25–40.00)	<0.01	5.00 (1.05–18.30)	41.25 (22.62–58.75)	3.00 (0.80–11.25)	0.28	<0.01
**Lung function values (Post bronchodilator)**								
FVC (%)	81.00 (66.00–92.50)	94.00 (85.00–106.00)	<0.01	72.00 (59.00–85.00)	62.00 (54.00–75.00)	95.00 (84.00–103.00)	<0.01	<0.01
FEV_1_ (%)	54.00 (34.00–67.00)	97.00 (87.00–107.75)	<0.01	77.00 (63.00–92.00)	67.00 (63.00–79.00)	98.00 (88.00–112.00)	<0.01	<0.01
FEV_1/_FVC (%)	51.00 (39.50–59.90)	80.50 (76.22–84.00)	<0.01	86.00 (81.95–91.00)	82.00 (80.00–91.05)	79.50 (75.60–82.50)	<0.01	0.07
DLco (%)	–	–	–	50.00 (33.00–68.50)	19.73 (12.80–27.30)	102.00 (89.00–114.00)	<0.01	<0.01

*Data presented with medians and quartiles (Q1 and Q3) for quantitative variables and percentages for qualitative variables. P-value obtained by Mann-Whitney U-test for continuous variables and χ^2^-test for categorical variables. p < 0.05. COPD-S, Tobacco-smoking patients with COPD; SWOC, smokers without COPD; IPF, Patients with Idiopathic Pulmonary Fibrosis; PAC, Pulmonary Aging Cohort subjects; CPFE, Patients with Combined Pulmonary Fibrosis and Emphysema Syndrome; M, Male; F, Female; BMI, Body Mass Index; S, Smokers; NS, Non-Smokers; TI, Tobacco index; FVC, Forced Vital Capacity; FEV_1_, Forced Expiratory Volume in the first second. DLco, Pulmonary diffusion capacity;*

* *p: Comparison IPF vs. PAC*;

** *p: Comparison: CPFE vs. PAC*.

The differences observed for the comparisons between groups of cases and controls in pulmonary function tests are expected since they are part of the diagnostic criteria to differentiate them. It should be noted that the CPFE patients had the lowest DL_CO_ levels in the cases' groups.

### Hardy-Weinberg Equilibrium

The Hardy-Weinberg equilibrium (HWE) was tested for the control group for each comparison. The rs2609255 and rs111521887 do not meet HWE in the SWOC group (*p* < 0.05). While for the PAC group, rs2736100, rs5743890, rs111521887 did not meet this criterion.

### Allele and Genotype Frequencies

In the COPD comparison group, no differences were found in allele frequencies in any of the included SNPs (Data presented in Table 2); however, when correcting for covariates (age, sex, BMI, and tobacco index), significant values were found for the *FAM13A* rs2609255 TG genotype OR = 1.60 (CI 95% = 1.09 – 2.35 *p* < 0.01), presenting an association with an increased risk for COPD.

**Table 2:** Allele and genotype frequencies of COPD-S vs. SWOC comparison.

**SNP/Gene**	**COPD-S** ***n*** **= 178 (%)**		**SWOC** ***n*** **= 367 (%)**		***p***	***p*** *	**OR**	**CI 95%**
rs2609255/*FAM13A*								
TT	80	(48.1)	168	(54.0)	0.22	0.01	0.79	0.54–1.15
TG	77	(46.3)	109	(35.0)	0.01	<0.01	1.60	1.09–2.35
GG	9	(5.6)	34	(11.0)	0.04	0.95	0.46	0.21–0.99
T	237	(71.4)	445	(72.8)	0.95	0.01	0.99	0.73–1.33
G	95	(28.6)	117	(27.2)			1.00	0.75–1.35
rs2736100/*TERT*								
AA	73	(41.2)	155	(42.2)	0.82	1	0.96	0.66–1.38
AC	77	(43.5)	162	(44.1)	0.84	0.83	0.96	0.67–1.38
CC	27	(15.3)	50	(13.7)	0.6	0.59	1.14	0.68–1.89
A	223	(62.9)	472	(61.7)	0.67	0.77	0.94	0.72–1.22
C	131	(37.1)	262	(38.3)			1.05	0.81–1.37
rs2076295/*DSP*								
TT	72	(41.1)	156	(42)	0.84	0.98	0.96	0.66–1.38
TG	76	(43)	165	(44.4)	0.81	0.98	0.95	0.66–1.37
GG	27	(15.9)	50	(13.6)	0.54	0.98	1.17	0.70–1.94
T	220	(62.8)	477	(64.2)	0.64	0.9	0.94	0.72–1.22
G	130	(37.2)	265	(35.8)			1.06	0.81–1.38
rs5743890/*TOLLIP*								
TT	140	(80.4)	303	(83.2)	0.46	0.92	0.84	0.53–1.33
CT	33	(18.9)	58	(15.9)	0.36	0.53	1.20	0.77–1.93
CC	1	(1)	5	(0.9)	0.41	0.66	0.41	0.04–3.59
T	313	(89.9)	664	(90.7)	0.68	0.65	0.91	0.59–1.40
C	35	(10.1)	68	(9.3)			1.09	0.71–1.67
rs111521887/*TOLLIP*								
CC	26	(14.8)	56	(15.3)	0.88	0.33	0.96	0.58–1.59
CG	149	(85.2)	307	(84.1)	0.75	0.39	1.08	0.65–1.78
GG	0	0	2	(0.6)	NA	NA	NA	NA
C	201	(57.4)	419	(57.3)	0.99	0.99	1.00	0.77–1.29
G	149	(42.6)	311	(42.7)			0.99	0.77–1.29

Genotypes and alleles frequencies were compared by χ^2^ test (p). Significant differences were demonstrated when p < 0.05. p^*^: value adjusted by age, sex, BMI, and tobacco index, by a binary logistic regression model. SNP, Single Nucleotide Polymorphism; COPD-S, Tobacco-smoking patients with COPD; SWOC, Smokers without COPD; OR, Odds ratio; CI, 95%, Confidence interval 95%; NA, Not apply.

Interestingly, the C allele of rs5743890 in the *TOLLIP* gene had the lowest frequency of all the SNPs included (9%).

Table 3 shows the comparison of allele and genotype frequencies for the IPF patients and PAC subjects. A significant difference in the frequencies of the G allele of the *FAM13A* rs2609255 was found, ~10% between cases and controls (OR = 1.69, CI 95% = 1.11–2.57, *p* = 0.01), in the same way, a significant difference was found between the frequency of the GG genotype with the highest presence in the case group (OR = 2.86, CI 95% = 1.26–7.06, *p* = 0.01) conferring a greater IPF susceptibility. Excitingly, this association remains significant after adjusting for covariates (*p* = 0.01). For rs2076295 in the preliminary analysis, a significant difference was found in the TT genotype that occurs more frequently in cases (OR = 1.79 CI 95% = 1.02–3.12, *p* = 0.03), granting a greater risk for IPF development; however, it does not remain significant after adjustment for covariates.

**Table 3:** Allele and genotype frequencies of IPF vs. PAC comparison.

**SNP/Gene**	**IPF** ***n*** **= 93 (%)**		**PAC** ***n*** **= 174 (%)**		***p***	***p*** *	**OR**	**CI 95%**
rs2609255/*FAM13A*								
TT	28	(39.4)	80	(51.4)	0.10	0.11	0.62	0.35–1.10
TG	30	(42.2)	67	(41.3)	0.95	0.26	0.98	0.55–1.73
GG	13	(18.4)	10	(7.3)	0.01	0.31	2.86	1.26–7.06
T	86	(60.5)	227	(72.3)	0.01	0.01	0.58	0.38–0.89
G	56	(39.5)	87	(27.7)			1.69	1.11–2.57
rs2736100/*TERT*								
AA	47	(57.3)	63	(42.5)	0.03	0.16	1.81	1.04–3.12
AC	29	(35.3)	55	(37.1)	0.78	0.91	0.92	0.52–1.62
CC	6	(7.4)	30	(20.4)	0.01	0.07	0.33	0.13–0.84
A	123	(75)	181	(61.1)	<0.01	<0.01	1.9	1.24–2.91
C	41	(25)	115	(38.9)			0.52	0.34–0.80
rs2076295/*DSP*								
TT	38	(53.5)	63	(37.7)	0.03	0.86	1.79	1.02–3.12
TG	24	(33.8)	82	(49.1)	0.02	<0.01	0.52	0.29–0.94
GG	9	(12.7)	22	(13.2)	0.91	<0.01	0.95	0.41–2.19
T	100	(70.4)	208	(62.2)	0.08	0.05	1.44	0.94–2.20
G	42	(26.6)	126	(37.8)			0.52	0.34–0.80
rs5743890/*TOLLIP*								
TT	57	(100)	37	(22.4)	NA	NA	NA	NA
TC	0	(0)	119	(72.1)				
CC	0	(0)	9	(5.5)				
T	114	(100)	193	(58.4)	NA	NA	NA	NA
C	0	(0)	137	(41.6)				
rs111521887/*TOLLIP*								
CC	22	(25.8)	18	(11.5)	<0.01	0.09	2.67	1.34–5.34
CG	63	(74.2)	138	(88.5)	<0.01	0.95	0.37	0.18–0.74
GG	0	(0)	0	(0)	NA	NA	NA	NA
C	107	(62.9)	174	(55.7)	0.12	<0.01	1.34	0.91–1.97
G	63	(37.1)	138	(44.3)			0.74	0.50–1.08

Genotype and allele frequencies were compared by χ^2^ test (p). Significant differences were demonstrated when p < 0.05. p^*^: value adjusted by age, sex, and tobacco index, by a binary logistic regression model. SNP, Single Nucleotide Polymorphism; IPF, Patients with Idiopathic Pulmonary Fibrosis; PAC, Pulmonary Aging Cohort subjects; OR, Odds ratio; CI, 95%, Confidence interval 95%; NA, Not apply.

For the CPFE comparison, statistically significant differences for rs2736100 were found, with a higher A allele frequency in controls, associated with a decreased risk for CPFE (OR = 0.24, CI 95% = 0.11–0.55, *p* < 0.01); while the C allele, with a higher frequency in cases, yielding an increased susceptibility to CPFE (OR = 4.02 CI 95% = 1.79–8.99, *p* < 0.01). Data are presented in Table 4.

**Table 4:** Allele and genotype frequencies of CPFE vs. PAC comparison.

**SNP/gene**	**CPFE**		**PAC**		***p***	***p*** ^*^	**OR**	**CI 95%**
	***n*** **= 16 (%)**		***n*** **= 174 (%)**					
rs2609255/*FAM13A*								
TT	6	(37.5)	80	(51.4)	0.43	0.99	0.57	0.20–1.66
TG	8	(50)	67	(41.3)	0.6	0.99	1.34	0.47–3.76
GG	2	(12.5)	10	(7.3)	0.3	0.99	2.1	0.41–10.54
T	20	(64)	227	(72.3)	0.3	0.3	0.64	0.30–1.38
G	12	(36)	87	(27.7)			1.55	0.72–3.32
rs2736100/*TERT*								
AA	1	(6.3)	63	(42.5)	0.04	0.99	0.08	0.01–0.69
AC	7	(43.7)	55	(37.1)	0.59	0.99	1.31	0.46–3.72
CC	8	(50)	30	(20.4)	0.01	0.99	3.93	1.36–11.33
A	9	(28.1)	181	(61.1)	<0.01	<0.01	0.24	0.11–0.55
C	23	(71.9)	115	(38.9)			4.02	1.79–8.99
rs2076295/*DSP*								
TT	7	(43.75)	63	(37.7)	0.78	0.99	1.28	0.45–3.61
TG	2	(12.5)	82	(49.1)	<0.01	0.99	0.14	0.03–0.67
GG	7	(43.75)	22	(13.2)	<0.01	0.99	5.12	1.73–15.16
T	16	(50)	208	(62.2)	0.18	0.91	0.6	0.29–1.25
G	16	(50)	126	(37.8)			1.65	0.79–3.14
rs111521887/*TOLLIP*								
CC	2	(12.5)	18	(11.5)	1	0.99	1.09	0.22–5.21
CG	14	(87.5)	138	(88.5)	1	0.99	0.91	0.19–4.34
GG	0	(0)	0	(0)	NA	NA	NA	NA
C	18	(56.2)	174	(55.7)	1	0.26	1.01	0.48–2.12
G	14	(43.7)	138	(44.3)			0.98	0.47–2.04

*Genotypes and alleles frequencies were compared by Fisher exact test (p). Significant differences were demonstrated when p < 0.05*.

* *p: value adjusted by age, sex, and tobacco index, by a binary logistic regression model. SNP, Single Nucleotide Polymorphism; CPFE, Patients with Combined Pulmonary Fibrosis and Emphysema Syndrome; PAC, Pulmonary Aging Cohort subjects; OR, Odds ratio; CI, 95%, Confidence interval 95%; NA, Not apply*.

### Genetic Association Models

Codominant, dominant, and recessive models were applied for significant SNPs in the IPF comparison (Tables 5, 6). The rs2609255 association remains in the codominant (OR = 3.29) and recessive (OR = 2.86) models. For rs2076295, the association remains significant when applying the dominant model (OR = 1.90). These models also were applied in the COPD and CPFE comparisons. The first one was without significant association for none of the SNPs evaluated (Supplementary Tables 2, 3). For CPFE comparison, the rs2736100 AA genotype association remains in the dominant model (OR = 0.08), also applying the recessive model, a significant association was found for the CC genotype (OR = 3.93). When the recessive model is applied, we found a significant association for the rs2076295 GG genotype (OR = 5.12), conferring an increased risk to CPFE (Supplementary Tables 4, 5).

**Table 5:** Codominant model of IPF vs. PAC comparison.

	**IPF (%)**	**PA (%)**	***p***	**OR**	**CI 95%**
rs2609255/*FAM13A*					
TT	39.4	51.4		1	Ref
TG	42.2	41.3	0.03	1.33	0.71–2.48
GG	18.4	7.3		3.29	1.29–8.38
rs2736100/*TERT*					
AA	57.3	42.5		1	Ref
AC	35.3	37.1	<0.01	0.7	0.39–1.27
CC	7.4	20.4		0.26	0.10–0.69
rs2076295/*DSP*					
TT	53.5	37.7		1	Ref
TG	33.8	49.1	0.09	0.48	0.26–0.89
GG	12.7	13.2		0.67	0.28–1.62
rs5743890/TOLLIP					
TT	100	22.4		1	Ref
TC	0	72.1	NA	NA	NA
CC	0	5.5			
rs111521887/*TOLLIP*					
CC	25.8	11.5		1	Ref
CG	74.2	88.5	NA	NA	NA
GG	0	0			

*Genotype frequencies were compared by the χ^2^-test (p). Data presented with percentage Significant differences were demonstrated when p < 0.05. SNP, Single Nucleotide Polymorphism; IPF, Patients with Idiopathic Pulmonary Fibrosis; PAC, Pulmonary Aging Cohort subjects; OR, Odds ratio; CI 95%, Confidence interval 95%; Ref, Reference; NA, Not apply*.

**Table 6:** Dominant and recessive model of IFP vs. PAC comparison.

**Model**	**IPF (%)**	**PAC (%)**	***p***	**OR**	**CI 95%**
rs2609255/*FAM13A*					
Dom					
TT	39.4	51.4	0.10	0.62	0.35–1.10
TG + GG	60.6	48.6		1.59	0.90–2.82
Rec					
TT + TG	81.6	92.7	0.01	0.3	0.12–0.73
GG	18.4	7.3		2.86	1.26–7.06
rs2736100/*TERT*					
Dom					
AA	57.9	42.5	0.03	1.81	1.04–3.12
AC + CC	42.7	57.5		0.55	0.31–0.95
Rec					
AA + AC	92.6	79.6	<0.01	3.22	1.27–8.10
CC	7.4	20.4		0.31	0.12–0.78
rs2076295/*DSP*					
Dom					
TT	53.5	37.7	0.03	1.9	1.08–3.33
TG + GG	46.5	62.3		0.52	0.30–0.92
Rec					
TT + TG	87.3	86.8	0.91	1.04	0.45–2.39
GG	12.7	13.2		0.95	0.41–2.19
rs5743890/*TOLLIP*					
Dom					
TT	100	22.4	NA	NA	NA
TC + CC	0	77.6		NA	NA
Rec					
TT + TC	100	94.5	NA	NA	NA
CC	0	5.5		NA	NA
rs111521887/*TOLLIP*					
Dom					
CC	25.8	11.5	<0.01	2.67	1.34–5.34
CG + GG	74.2	85.5		0.37	0.18–0.74
Rec					
CC + CG	100	100	NA	NA	NA
GG	0	0		NA	NA

*Genotype frequencies were compared by χ^2^-test (p). Data presented with percentage Significant differences were demonstrated when p < 0.05. SNP, Single Nucleotide Polymorphism; IPF, Patients with Idiopathic Pulmonary Fibrosis; PAC, Pulmonary Aging Cohort subjects; Dom, Dominant model; Rec, Recessive model; OR, Odds ratio; CI, 95%, Confidence interval 95%; NA, Not apply*.

### Univariate and Multivariate Logistic Regression Model

Univariate and multivariate logistic regression analyses were performed for the recessive model of rs2609255 (*FAM13A*) in the IPF comparison, where a possible relationship of the genotype with clinical variables associated with prognosis or development may be involved. The variables to consider were the tomographic pattern, BAL cell count, and pulmonary function tests (FVC and DL_CO_); however, no relationship was found between patients' genotype and the clinical variables (Supplementary Table 6).

## Discussion

In this case-control study, we investigated the potential associations of rs2609255 (*FAM13A*), rs2736100 (*TERT*), rs2076295 (*DSP*) rs5743890, and rs111521887 (*TOLLIP*) with IPF, COPD, and CPFE syndrome in a Mexican-mestizo population. We found that the rs2609255/G allele (*FAM13A*) has an independent effect on the risk for IPF in our population. In addition, an association with the rs2076295/TT genotype (*DSP*) was significantly associated with increased susceptibility for IPF. The rs2609255/TG genotype (*FAM13A*) is associated with COPD susceptibility. For the CPFE group, we also found a significant association of rs2076295/GG genotype (*DSP*) and rs2736100/CC genotype (*TERT*) to a higher risk for CPFE syndrome.

The prominent and distinctive characteristic of the CPFE syndrome is the marked loss of gas diffusion capacity, represented by DL_CO_ values (25), which correlates with our results when comparing the DL_CO_ levels among the three cases' groups.

Regarding clinical and demographic variables of patients' groups and control subjects, we observed that the CPFE patients are the group that presents the lowest DL_CO_, FEV_1_, and FVC values when were compared with the IPF patients and the PAC subjects. Interestingly, despite the decrease in FEV_1_ and FVC, the FEV_1_/FVC ratio was maintained at >70%, another characteristic previously reported in CPFE patients (9, 13, 25).

Multiple studies have identified variants associated with susceptibility for both COPD and IPF (17, 26). However, there are few genetic association studies reported for CPFE. Multiple GWAS have identified common single-nucleotide variants with a MAF>5% associated with IPF in several genes that play a crucial role in different pathways related to disease pathogenesis, among which are *MUC5B, FAM13A, DSP, TOLLIP, TERT, TERC, MDGA2, SPPL2C* (17, 26).

*FAM13A* (family with sequence similarity 13, member A) is a gene allocated on cytogenetic band 22 of the long arm of chromosome 4, expressed in multiple tissues and cells, such as type 2 epithelial cells and airway macrophages. However, little is known about its biological function (27, 28). Genetic variants in *FAM13A* have been associated with both COPD and IPF (17, 27, 29, 30). In 2016, Hirano et al. reported in a Japanese cohort that the association with IPF susceptibility was associated with the rs2609255 G allele; furthermore, they demonstrated that in the recessive, dominant, and additive genetic association models, the GG genotype was also associated with an IPF increased risk (27). Our IPF group showed higher frequencies of the G allele and the GG genotype of rs2609255, finding an association with the increased risk for IPF. However, when adjusting for covariates, only the G allele remained significantly associated. On the other hand, when applying the co-dominant and recessive association models, the GG genotype remained associated, conferring a higher risk for IFP.

Different studies have also described that *FAM13A* is associated with COPD susceptibility. Wang et al. evaluated 5 SNPs (rs7671167, rs2869966, rs2869967, rs2045517, and rs6830970), showing that they were associated with lower FEV_1_/FVC values and that rs767167 conferred a higher risk for COPD. On the other hand, Zhang et al. reported an association between rs17014601 and COPD in additive, heterozygous, and dominant models (31). No association between COPD and rs2609255 has been previously reported; in our case, when performing the analysis of allele frequencies and genotypes in the COPD-S and SWOC group; after correction for covariates (age, sex, TI, and BMI), we found that the TG genotype was associated with an increased risk for developing COPD. This variant in the SWOC group does not comply with the HWE; however, the HWE is not met in multiple populations. The Mexican mestizo population has a very diverse genetic variability due to years of genetic recombination with other populations such as European, Amerindian, and Asian, so probably several SNPs do not behave in the same way previously described in the literature (32, 33).

*FAM13A* is associated with the Wnt signaling pathway; in a COPD animal model, it promotes β-catenin degradation and decreases Wnt signaling; while in fibrosis, a mechanism by which *FAM13A* may be conditioning susceptibility has not been proposed. In IPF patients, there is an increase in FAM13A protein, inducing fibroblast migration (27), which suggests that *FAM13A* plays an important role in the IPF pathogenesis.

In the genotype frequencies crude analysis, another significant association was found within the IPF patients group. The rs2076295 TT genotype of *DSP* is associated with increased susceptibility to IPF. Previous studies showed an association between intronic variants of the *DSP* gene with IPF. Mathai et al. reported that the rs2076295 G allele in intron 5 confers a higher risk in the Caucasian population; also that the minor allele was associated with a lower *DSP* expression in the lung (34). In our IPF patients group, the TT genotype is associated with higher risk, and when applying the dominant model, this association remains significant. The *DSP* gene encodes for desmoplakin, a binding protein present in desmosomes; the rs2076295 is considered a binding site for the transcription factor PU.1 involved in macrophage activation and airway remodeling (34, 35).

Alterations in telomeres and their length are etiological factors of multiple diseases. For example, rs2736100 of the *TERT* gene, a component of the telomerase enzyme, is strongly associated with telomere length and increased susceptibility for IPF (36). Previous studies have shown that the A allele of this variant is associated with shorter telomere length in peripheral blood leukocytes (37, 38). On the other hand, this same variant is associated with increased telomere length in patients with lung cancer (37).

Mutations in *TERT* have also been associated with the development of emphysema and COPD. For example, Ding et al. (38) reported an increased risk for COPD with several SNPs, including rs10069690, rs2853677, and rs2853676. In addition, Stanley et al. (39) found mutations in *TERT* present in female smokers with severe emphysema and a tendency to the presence of pneumothorax.

Our study found an association of the C allele of rs2736100 of *TERT* and the CC genotype in the recessive and codominant model, giving increased susceptibility for CPFE; this is the first significant association between rs2736100 and CPFE syndrome described.

It should be noted that in the IPF group, this variant is associated when comparing allele frequencies. The A allele is associated with increased risk for IPF while the C allele with CPFE risk; we could speculate then that there is a differential genetic profile between these two conditions.

CPFE syndrome is a relatively new and poorly described entity; there are still many unknowns about its etiopathogenesis. The participation of immunological, inflammatory, and genetic factors has been proposed in several reviews; however, being such a rare entity, few studies have been reported to date. Although we have a limited sample size for this entity, we could observe a difference in the behavior of the SNPs analyzed in each disease, conferring risk for one and not for the other two.

Patients with IPF have a poor prognosis, with a maximum survival of 5 years from diagnosis. Previous studies have searched for relationships between the genetic variants analyzed and clinical variables associated with a worse prognosis in patients, such as DL_CO_ values, the extension of the tomographic pattern, and LBA cell count. For example, in IPF patients, Wang and collaborators (36) reported that the CC + CT model of rs868903 of *MUC5B* was associated with shorter survival, shorter telomere length, and higher tomographic pattern (honeycomb) extension. In addition, Bonella et al. reported an association between the C allele of rs5743890 of the *TOLLIP* gene and lower survival and disease progression. On the other hand, the T allele of rs2609255 is associated with lower DL_CO_ levels and lower survival (40). We performed univariate and multivariate logistic regression models looking for a relation between the TT + TG vs. GG model and variables associated with prognosis or development (tomographic pattern, LBA cell count, and spirometry values); however, no significant association was identified, possibly due to our sample size. Therefore, additional investigations should be done to identify factors associated with a worse prognosis.

The well-defined cases' groups (clinical and tomographically) and the 2:1 case-controls ratio are some of our research strengths. Besides, the PAC group is ideal for making comparisons since it comprises aging subjects without evidence of lung disease, avoiding potential confounding factors. In addition, there are no studies where the genetic variants of both entities involved in CPFE syndrome are compared.

Our study is not free of limitations. For example, the sample size was small compared to other studies such as GWAS. All the participants were recruited from a single center, and the CPFE recruitment was suspended due to the COVID-19 lockdown. We also had limitations regarding the tomographic data since only the diagnostic tomography was considered to analyze genotypes and variables associated with a worse prognosis since several patients did not have more than one tomography in the follow-up. Besides, we only consider the presence of the usual interstitial pneumonia pattern (honeycomb) as typical or not, without considering its extension for the analysis. More studies are needed with a more significant number of patients and to be able to corroborate the previous findings.

In summary, the rs2736100 C allele of *TERT* is associated with decreased IPF risk and confers an increased risk for CPFE; besides, the A allele is also associated with IPF increased susceptibility, while the CPFE comparison provides a protection factor (OR <1.0). Also, the rs2076295 TT genotype of *DSP* is associated with increased IPF risk, while the GG genotype is associated with CPFE susceptibility. The rs2609255 G allele and GG genotype of *FAM13A* are associated with IPF susceptibility, while the TG genotype is present in patients with emphysema and provides COPD susceptibility. These findings support the hypothesis that there is a differential genomic profile between COPD patients with emphysema, IPF, and CFPE. Our findings point to different molecular pathways between the three diseases and the role of SNP variants in the pathogenesis of CPFE, which could even be used as genetic markers to differentiate these patients.

In conclusion, we described for the first time that some polymorphisms in the *TERT* and *DSP* genes are associated with a higher risk for CPFE. Interestingly, one of these SNPs is associated with reduced risk for IPF while not associated with COPD. These findings suggest the existence of a differential genomic profile between COPD, IPF, and CPFE syndrome. However, more studies are needed to elucidate the role of genetic variants in the development of CPFE.

## Data Availability Statement

The datasets presented in this study can be found in online repositories. The names of the repository/repositories and accession number(s) can be found at: https://www.ncbi.nlm.nih.gov/, SCV001712126-SCV001712130; https://www.ncbi.nlm.nih.gov/, SCV001712131-SCV001712135; https://www.ncbi.nlm.nih.gov/, SCV001749000-SCV001749003.

## Ethics Statement

The studies involving human participants were reviewed and approved by Institutional Committees for Research, Ethics in Research, and Biosecurity of the INER (approval number: C09-19 and C39-14). The patients/participants provided their written informed consent to participate in this study.

## Author Contributions

RF-V, IB-R, and AR-V: conceptualization. JG-V, MAP-G, and EA-O: data curation and methodology. JG-V, GP-R, EA-O, and RF-V: formal analysis and writing—original draft. RF-V and IB-R: funding acquisition and resources. GP-R, RdJH-Z, MM, IB-R, and RF-V: investigation. GP-R, IB-R, AR-V, RdJH-Z, and RF-V: project administration. JG-V, EA-O, and GP-R: software. GP-R, IB-R, MM, and RF-V: supervision. RF-V: visualization. JG-V, EA-O, and RF-V: writing—review and editing. All authors contributed to the article and approved the submitted version.

## Conflict of Interest

The authors declare that the research was conducted in the absence of any commercial or financial relationships that could be construed as a potential conflict of interest.

## Publisher's Note

All claims expressed in this article are solely those of the authors and do not necessarily represent those of their affiliated organizations, or those of the publisher, the editors and the reviewers. Any product that may be evaluated in this article, or claim that may be made by its manufacturer, is not guaranteed or endorsed by the publisher.

## References

[1] Lõpez-Campos JL Tan W Soriano JB. Global burden of COPD. Respirology. (2016) 21:14–23. 10.1111/resp.12660 26494423

[2] Soriano JB Abajobir AA Abate KH Abera SF Agrawal A Ahmed MB. Global, regional, and national deaths, prevalence, disability-adjusted life years, and years lived with disability for chronic obstructive pulmonary disease and asthma, 1990–2015: a systematic analysis for the Global Burden of Disease Study 2015. Lancet Respir Med. (2017) 5:691–706. 10.1016/S2213-2600(17)30293-X 28822787 PMC5573769

[3] Lin H Jiang S. Combined pulmonary fibrosis and emphysema (CPFE): an entity different from emphysema or pulmonary fibrosis alone. J Thorac Dis. (2015) 7:767−79. 25973246 10.3978/j.issn.2072-1439.2015.04.17 PMC4419325

[4] Papaioannou AI Kostikas K Manali ED Papadaki G Roussou A Kolilekas L. Combined pulmonary fibrosis and emphysema: the many aspects of a cohabitation contract. Respir Med. (2016) 117:14–26. 10.1016/j.rmed.2016.05.005 27492509

[5] Portillo K Morera J. Combined pulmonary fibrosis and emphysema syndrome: a new phenotype within the spectrum of smoking-related interstitial lung disease. Pulmonary Med. (2012) 2012:1–8. 10.1155/2012/867870 22448331 PMC3289935

[6] Amariei DE Dodia N Deepak J Hines SE Galvin JR Atamas SP. Combined pulmonary fibrosis and emphysema: pulmonary function testing and a pathophysiology perspective. Medicina. (2019) 55:1–14. 10.3390/medicina55090580 31509942 PMC6780454

[7] Jankowich MD Rounds SIS. Combined pulmonary fibrosis and emphysema syndrome: a review. Chest. (2012) 141:222–31. 10.1378/chest.11-1062 22215830 PMC3251269

[8] Wakwaya Y Brown KK. Idiopathic pulmonary fibrosis: epidemiology, diagnosis and outcomes. Am J Med Sci. (2019) 357:359–69. 10.1016/j.amjms.2019.02.013 31010461

[9] Zhang L Zhang C Dong F Song Q Chi F Liu L. Combined pulmonary fibrosis and emphysema: a retrospective analysis of clinical characteristics, treatment and prognosis. BMC Pulm Med. (2016) 16:1–8. 10.1186/s12890-016-0300-7 27809901 PMC5093954

[10] Pérez-Rubio G Córdoba-Lanús E Cupertino P Cartujano-Barrera F Campos MA Falfán-Valencia R. Role of Genetic Susceptibility in Nicotine Addiction and Chronic Obstructive Pulmonary Disease. (2019). Available online at: www.clinicalandtranslationalinvestigation.com (accessed July 19, 2021). 10.24875/RIC.18002617 30810540

[11] Hernández-Montoya J Pérez-Ramos J Montaño M Ramírez-Venegas A Sansores RH Pérez-Rubio G. Genetic polymorphisms of matrix metalloproteinases and protein levels in chronic obstructive pulmonary disease in a Mexican population. Biomark Med. (2015) 9:979–88. 10.2217/bmm.15.75 26439471

[12] Dias OM Baldi BG Costa AN Carvalho CRR. Combinação de fibrose pulmonar e enfisema: Uma doença cada vez mais reconhecida. J Brasil Pneumol. (2014) 40:304−12. 10.1590/s1806-37132014000300014 25029654 PMC4109203

[13] Hage R Gautschi F Steinack C Schuurmans MM. Combined pulmonary fibrosis and emphysema (CPFE) clinical features and management. Int J Chron Obstruct Pulmon Dis. (2021) 16:167–77. 10.2147/COPD.S286360 33536752 PMC7850450

[14] Kinjo T Kitaguchi Y Droma Y Yasuo M Wada Y Ueno F. The Gly82Ser mutation in AGER contributes to pathogenesis of pulmonary fibrosis in combined pulmonary fibrosis and emphysema (CPFE) in Japanese patients. Sci Rep. (2020) 10:1–10. 10.1038/s41598-020-69184-8 32732977 PMC7393115

[15] Xu L Bian W Gu XH Shen C. Genetic polymorphism in matrix metalloproteinase-9 and transforming growth factor-β1 and susceptibility to combined pulmonary fibrosis and emphysema in a Chinese population. Kaohsiung J Med Sci. (2017) 33:124–9. 10.1016/j.kjms.2016.12.004 28254114 PMC11916232

[16] Wang B Liang B Yang J Xiao J Ma C Xu S. Association of FAM13A polymorphisms with COPD and COPD-related phenotypes in Han Chinese. Clin Biochem. (2013) 46:1683–8. 10.1016/j.clinbiochem.2013.07.013 23891779

[17] Fingerlin TE Murphy E Zhang W Peljto AL Brown KK Steele MP. Genome-wide association study identifies multiple susceptibility loci for pulmonary fibrosis. Nat Genet. (2013) 45:613–20. 10.1038/ng.2609 23583980 PMC3677861

[18] Buendía-Roldan I Fernández-Plata R Valdes-Bartolo A Mejia M Jaramillo LE Martínez-Briseño D. Determination of the phenotypic age in residents of Mexico City: effect of accelerated ageing on lung function and structure. ERJ Open Res. (2020) 6:1–10. 10.1183/23120541.00084-2020 32864379 PMC7445116

[19] Selman M Buendía-Roldán I Pardo A. Aging and Pulmonary Fibrosis. Available online at: www.permanyer.com 27103043

[20] Pérez-Padilla R Valdivia G Muiño A López MV Márquez MN De Oca MM. Valores de referencia espirométrica en 5 grandes ciudades de Latinoamérica para sujetos de 40 o más años de edad. Arch Bronconeumol. (2006) 42:317–25. 10.1157/13090581 16945261

[21] Raghu G Remy-Jardin M Myers JL Richeldi L Ryerson CJ Lederer DJ. American Thoracic Society documents diagnosis of idiopathic pulmonary fibrosis an official ATS/ERS/JRS/ALAT clinical practice guideline. Am J Respir Crit Care Med. (2018) 198:44–68. 10.1164/rccm.201807-1255ST 30168753

[22] RStudio Team. RStudio | Open Source & Professional Software for Data Science Teams. RStudio (2015). Available online at: https://www.rstudio.com/ (accessed May 18, 2021).

[23] CDC. Epi Info^TM^| CDC. Centers for Disease Control and Prevention (2018). p. 1. Available online at: https://www.cdc.gov/epiinfo/esp/es_index.html (accessed May 18, 2021).

[24] PLINK. PLINK_Whole genome association analysis toolset. Am J Human Genet. (2017) 81:559–75. 10.1086/519795 17701901 PMC1950838

[25] Ryerson CJ Hartman T Elicker BM Ley B Lee JS Abbritti M. Clinical features and outcomes in combined pulmonary fibrosis and emphysema in idiopathic pulmonary fibrosis. Chest. (2013) 144:234–40. 10.1378/chest.12-2403 23370641

[26] Noth I Zhang Y Ma S-F Flores C Barber M Huang Y. Genetic Variants Associated With Idiopathic Pulmonary Fibrosis Susceptibility and Mortality: A Genome-Wide Association Study. Available online at: http://www.lung-genomics.org (accessed April 30, 2021). 10.1016/S2213-2600(13)70045-6 PMC3894577 24429156

[27] Hirano C Ohshimo S Horimasu Y Iwamoto H Fujitaka K Hamada H. FAM13A polymorphism as a prognostic factor in patients with idiopathic pulmonary fibrosis. Respir Med. (2017) 123:105–9. 10.1016/j.rmed.2016.12.007 28137485

[28] Liang C Li A Haidar S Raza A Khan R Wang X. The Molecular Characteristics of the FAM13A Gene and the Role of Transcription Factors ACSL1 and ASCL2 in Its Core Promoter Region. Available online at: www.mdpi.com/journal/genes 10.3390/genes10120981 PMC6947481 31795267

[29] Jiang Z Lao T Qiu W Polverino F Gupta K Guo F. A chronic obstructive pulmonary disease susceptibility gene, FAM13A, regulates protein stability of β-catenin. Am J Respir Crit Care Med. (2016) 194:185–97. 10.1164/rccm.201505-0999OC 26862784 PMC5003213

[30] Cho MH Boutaoui N Klanderman BJ Sylvia JS Ziniti JP Hersh CP. Variants in FAM13A are associated with chronic obstructive pulmonary disease. Nat Genet. (2010) 42:200–2. 10.1038/ng.535 20173748 PMC2828499

[31] Zhang Y Qiu J Zhang P Zhang J Jiang M Ma Z. Genetic variants in FAM13A and IREB2 are associated with the susceptibility to COPD in a Chinese rural population: a case-control study. Int J COPD. (2018) 13:1735–45. 10.2147/COPD.S162241 29872291 PMC5973397

[32] Pérez-Rubio G Silva-Zolezzi I Fernández-López JC Camarena Á Velázquez-Uncal M Morales-Mandujano F. Genetic variants in IL6R and ADAM19 are associated with COPD severity in a Mexican Mestizo population. COPD J Chronic Obstr Pulm Dis. (2016) 13:610–5. 10.3109/15412555.2016.1161017 27078193

[33] Ambrocio-Ortiz E Pérez-Rubio G Abarca-Rojano E Montaño M Ramos C Hernández-Zenteno RDJ. Influence of proinflammatory cytokine gene polymorphisms on the risk of COPD and the levels of plasma protein. Cytokine. (2018) 111:364–70. 10.1016/j.cyto.2018.09.017 30296713

[34] Mathai SK Pedersen BS Smith K Russell P Schwarz MI Brown KK. Desmoplakin variants are associated with idiopathic pulmonary fibrosis. Am J Respir Crit Care Med. (2016) 193:1151–60. 10.1164/rccm.201509-1863OC 26669357 PMC4872666

[35] Kim W Cho MH Sakornsakolpat P Lynch DA Coxson HO Tal-Singer R. DSP variants may be associated with longitudinal change in quantitative emphysema. Respir Res. (2019) 20:1–10. 10.1186/s12931-019-1097-8 31324189 PMC6642569

[36] Wang H Zhuang Y Peng H Cao M Li Y Xu Q. The relationship between MUC5B promoter, TERT polymorphisms and telomere lengths with radiographic extent and survival in a Chinese IPF cohort. Sci Rep. (2019) 9:1–7. 10.1038/s41598-019-51902-6 31653936 PMC6814782

[37] Moore BB Flores C Canaria Rafael Clavijo F Yong Huang S Snetselaar R M van Oosterhout MF. Telomerase reverse transcriptase polymorphism rs2736100: a balancing act between cancer and non-cancer disease, a meta-analysis. Front Med (Lausanne). (2018) 5:1–10. 10.3389/fmed.2018.00041 29536006 PMC5835035

[38] Ding Y Li Q Wu C Wang W Zhao J Feng Q. TERT gene polymorphisms are associated with chronic obstructive pulmonary disease risk in the Chinese Li population. Mol Genet Genomic Med. (2019) 7:1–8. 10.1002/mgg3.773 31270965 PMC6687861

[39] Stanley SE Chen JJL Podlevsky JD Alder JK Hansel NN Mathias RA. Telomerase mutations in smokers with severe emphysema. J Clin Invest. (2015) 125:563–70. 10.1172/JCI78554 25562321 PMC4319417

[40] Bonella F Campo I Zorzetto M Boerner E Ohshimo S Theegarten D. Potential clinical utility of MUC5B und TOLLIP single nucleotide polymorphisms (SNPs) in the management of patients with IPF. Orphanet J Rare Dis. (2021) 16:1–9. 10.1186/s13023-021-01750-3 33639995 PMC7913255

