# Supplementary material for: Differential Genomic Profile in TERT, DSP, and FAM13A Between COPD Patients With Emphysema, IPF, and CPFE Syndrome
Source: Front Med (Lausanne). 2021 Aug 19;8:725144. doi: 10.3389/fmed.2021.725144 (PMC8416604; doi:10.3389/fmed.2021.725144)
Supplement: Supplementary file 1 [file Table_1.docx]

Supplementary Material

# Supplementary Figure


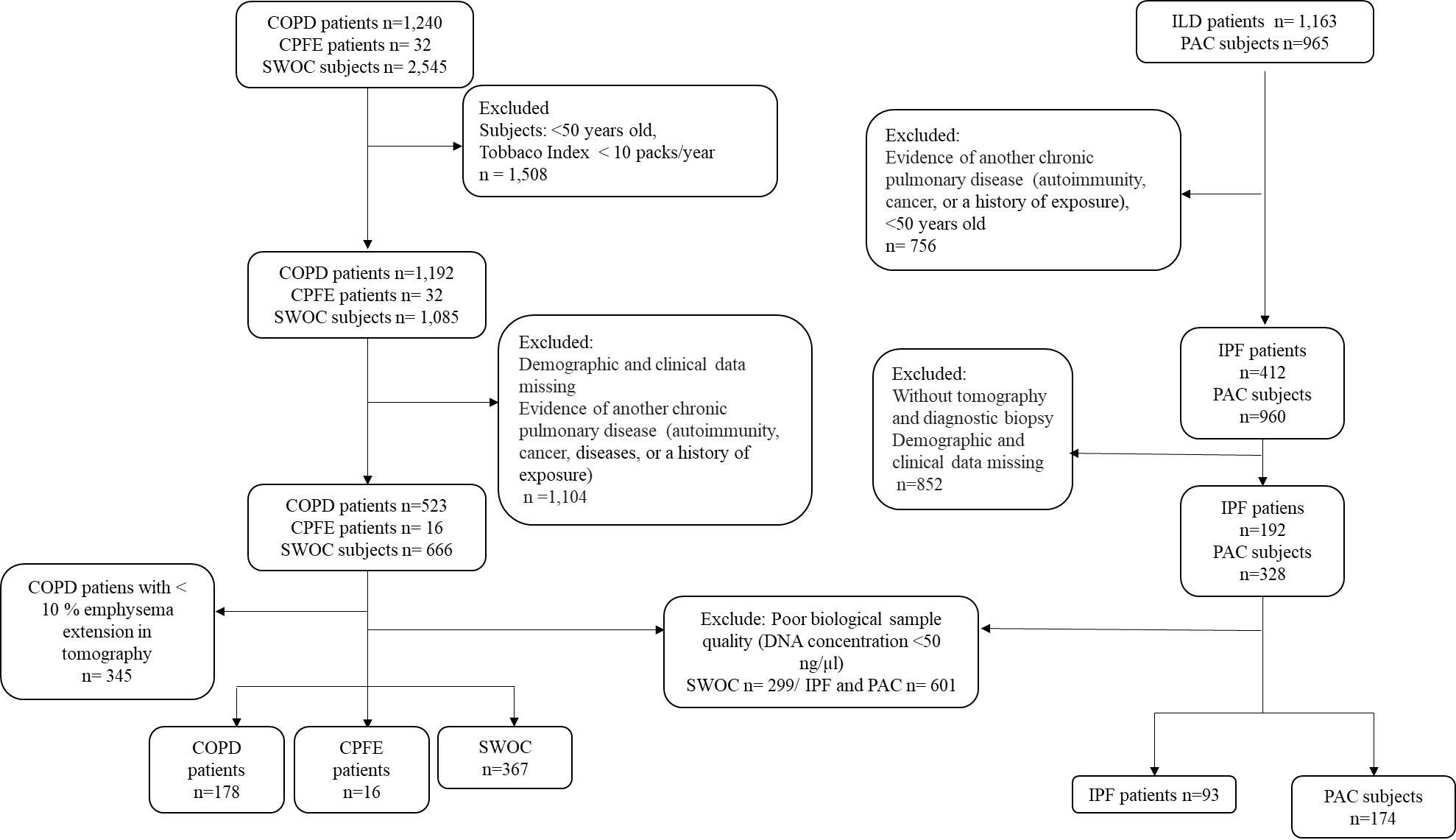


**Supplementary Figure 1.** Show selection process of patients and control subjects; Chronic Obstructive Disease (COPD), Combined Pulmonary Fibrosis and Emphysema Syndrome (CPFE)), Smokers without COPD (SWOC), Interstitial Lung Diseases (ILD), Idiopathic Pulmonary Fibrosis (IPF), Pulmonary Aging Cohort (PAC)

# Supplementary Tables

**Supplementary Table 1. Molecular data of SNPs evaluated.**

| **Gene** | **SNP** | **Chr** | **Change** | **1000 genomes** | **MAF(MXL)** | **pHWE*** | **pHWE**** |  |
| --- | --- | --- | --- | --- | --- | --- | --- | --- |
|  |  |  |  |  |  |  |  |  |
| *FAM13A* | rs2609255 | 4 | T>G | 0.34 | 0.258 | 0.017 | 0.694 |  |
| *TERT* | rs2736100 | 5 | A>C | 0.48 | 0.438 | 0.431 | 0.009 |  |
| *DSP* | rs2076295 | 6 | T>G | 0.43 | 0.43 | 0.571 | 0.623 |  |
| *TOLLIP* | rs5743890 | 11 | T>C | 0.04 | 0.086 | 0.22 | <0.05 |  |
|  | rs111521887 | 11 | C>G | 0.07 | 0.086 | <0.05 | <0.05 |  |

Single nucleotide polymorphism (SNP), Chromosome (Chr), Minor allele frequency (MAF), Mexican Ancestry in Los Angeles California (MXL) from 1000 genomes, Hardy-Weinberg equilibrium (pHWE). pHWE * (SWOC subjects), pHWE ** (PAC subjects); p> 0.05.

**Supplementary Table 2. Codominant model of COPD-S *vs*. SWOC comparison**

| **SNP/Gene** | **COPD-S (%)** | **SWOC (%)** | **p** | **OR** | **CI 95%** |
| --- | --- | --- | --- | --- | --- |
| rs2609255 /*FAM13A* | | | | | |
| TT | 48.1 | 54.0 |  | 1 | Ref |
| TG | 46.3 | 350. | 0.95 | 1.48 | 0.99-2.20 |
| GG | 5.6 | 11.0 |  | 0.55 | 0.25-1.21 |
| rs2736100/ *TERT* | | | | | |
| AA | 41.2 | 42.2 |  | 1 | Ref |
| AC | 43.5 | 44.1 | 0.68 | 1.00 | 0.68-1.48 |
| CC | 15.3 | 13.7 |  | 1.14 | 0.66-1.97 |
| rs2076295/ *DSP* | | | | | |
| TT | 41.1 | 42 |  | 1 | Ref |
| TG | 43 | 44.4 | 0.65 | 0.99 | 0.67-1.47 |
| GG | 15.9 | 13.6 |  | 1.17 | 0.67-2.01 |
| rs5743890/ *TOLLIP* | | | | | |
| TT | 80.4 | 83.2 |  | 1 | Ref |
| TC | 18.9 | 15.9 | 0.77 | 1.19 | 0.74-1.91 |
| CC | 1 | 0.9 |  | 0.42 | 0.04-3.63 |
| rs111521887/ *TOLLIP* | | | | | |
| CC | 14.8 | 15.3 |  | 1 | Ref |
| CG | 85.2 | 84.1 | NA | NA | NA |
| GG | 0 | 0.6 |  |  |  |

Genotype frequencies were compared by χ^2^ test (p). Data presented with percentage Significant differences were demonstrated when p <0.05. Single Nucleotide Polymorphism (SNP); Tobacco-smoking patients with COPD (COPD-S); Smokers without COPD, (SWOC); Odds ratio (OR); Confidence interval 95 % (CI, 95%); Reference (Ref); Not apply (NA).

**Supplementary Table 3. Dominant and Recessive Model of COPD *vs.* SWOC comparison**

| **Model** | **COPD-S**  **%** | **SWOC**  **%** | **p** | **OR** | **CI 95%** |
| --- | --- | --- | --- | --- | --- |
| rs2609255/*FAM13A* | | | | | |
| Dom |  |  |  |  |  |
| TT | 48.1 | 54 | 0.22 | 0.79 | 0.54-1.15 |
| TG+ GG | 51.9 | 46 |  | 1.26 | 0.86-1.84 |
| Rec |  |  |  |  |  |
| TT+TG | 94.4 | 89 | 0.66 | 2.14 | 1.00-4.58 |
| GG | 5.6 | 11 |  | 0.46 | 0.21-0.99 |
| rs2736100/*TERT* | | | | | |
| Dom |  |  |  |  |  |
| AA | 41.2 | 42.2 | 0.82 | 0.96 | 0.66-1.38 |
| AC+CC | 58.8 | 57.8 |  | 1.04 | 0.72-1.49 |
| Rec |  |  |  |  |  |
| AA+AC | 84.7 | 86.3 | 0.60 | 0.87 | 0.52-1.45 |
| CC | 15.3 | 13.7 |  | 1.14 | 0.68-1.89 |
| rs2076295/*DSP* | | | | | |
| Dom |  |  |  |  |  |
| TT | 41.1 | 42 | 0.84 | 0.96 | 0.66-1.38 |
| TG+ GG | 58.9 | 58 |  | 1.03 | 0.72-1.49 |
| Rec |  |  |  |  |  |
| TT+TG | 84.1 | 86.4 | 0.63 | 0.85 | 0.51-1.41 |
| GG | 15.9 | 13.6 |  | 1.17 | 0.70-1.94 |
| rs5743890/*TOLLIP* | | | | | |
| Dom |  |  |  |  |  |
| TT | 80.4 | 83.2 | 0.51 | 0.85 | 0.53-1.35 |
| TC+CC | 19.6 | 16.8 |  | 1.16 | 0.73-1.85 |
| Rec |  |  |  |  |  |
| TT+TC | 99.3 | 99.1 | 0.41 | 2.39 | 0.27-20.66 |
| CC | 0.7 | 0.9 |  | 0.41 | 0.04-3.59 |
| rs111521887/*TOLLIP* | | | | | |
| Dom |  |  |  |  |  |
| CC | 14.8 | 15.3 | 0.88 | 0.96 | 0.58-1.59 |
| CG+GG | 85.2 | 84.7 |  | 1.03 | 0.62-1.72 |
| Rec |  |  |  |  |  |
| CC+CG | 100 | 99.4 | NA | NA | NA |
| GG | 0 | 0.6 |  | NA | NA |

Genotype frequencies were compared by χ^2^ test (p). Data presented with percentage Significant differences were demonstrated when p <0.05. Single Nucleotide Polymorphism (SNP); Tobacco-smoking patients with COPD (COPD-S); Smokers without COPD, (SWOC); Dominant model (Dom); Recessive model (Rec); Odds ratio (OR); Confidence interval 95 % (CI 95%); Not apply (NA).

**Supplementary Table 4. Codominant model of CPFE *vs.* PAC comparison.**

| **Model** | **CPFE**  **%** | **PAC**  **%** | **p** | **OR** | **CI** |
| --- | --- | --- | --- | --- | --- |
| rs2609255*/FAM13A* | | | | | |
| TT | 37.5 | 51.4 |  | 1 | Ref. |
| TG | 50.0 | 41.3 | 0.25 | 1.66 | 0.54-5.06 |
| GG | 12.5 | 7.3 |  | 2.36 | 0.41-13.37 |
| rs2736100/*TERT* | | | | | |
| AA | 6.3 | 42.5 |  | 1 | Ref. |
| AC | 43.7 | 37.1 | <0.01 | 8.01 | 0.95-67.22 |
| CC | 50.0 | 20.4 |  | 16.8 | 2.00-140.5 |
| rs2076295/*DSP* | | | | | |
| TT | 43.7 | 37.7 |  | 1 | Ref. |
| TG | 12.5 | 49.1 | 0.18 | 0.21 | 0.04-1.09 |
| GG | 43.7 | 13.2 |  | 2.86 | 0.90-9.08 |
| rs111521887/*TOLLIP* | | | | | |
| CC | 12.5 | 11.5 |  | 1 | Ref |
| CG | 87.5 | 88.5 | NA | NA | NA |
| GG | 0 | 0 |  |  |  |

Genotype frequencies were compared by Fisher exact test (p). Data presented with percentage Significant differences were demonstrated when p <0.05. Single Nucleotide Polymorphism (SNP); Patients with Combined Pulmonary Fibrosis and Emphysema Syndrome (CPFE); Pulmonary Aging Cohort subjects (PAC); Odds ratio (OR); Confidence interval 95 % (CI 95%); Reference (Ref); Not apply (NA).

**Supplementary Table5. Dominant and Recessive Model of CPFE *vs.* PAC comparison.**

| **Model** | **CPFE**  **%** | **PAC**  **%** | **p** | **OR** | **CI, 95 %** |
| --- | --- | --- | --- | --- | --- |
| rs2609255/*FAM13A* | | | | | |
| Dom |  |  |  |  |  |
| TT | 37.5 | 51.4 | 0.30 | 0.57 | 0.20-1.66 |
| TG+ GG | 62.5 | 48.6 |  | 1.73 | 0.60-4.99 |
| Rec |  |  |  |  |  |
| TT+TG | 87.5 | 92.7 | 0.35 | 0.47 | 0.09-2.39 |
| GG | 12.5 | 7.3 |  | 1.82 | 0.36-9.19 |
| rs2736100/*TERT* | | | | | |
| Dom |  |  |  |  |  |
| AA | 6.3 | 42.5 | <0.01 | 0.08 | 0.01-0.69 |
| AC+CC | 93.7 | 57.5 |  | 11.11 | 1.43-86.38 |
| Rec |  |  |  |  |  |
| AA+AC | 50 | 79.6 | 0.01 | 0.26 | 0.08-0.73 |
| CC | 50 | 20.4 |  | 3.93 | 1.36-11.33 |
| rs2076295/*DSP* | | | | | |
| Dom |  |  |  |  |  |
| TT | 43.75 | 37.7 | 0.78 | 1.28 | 0.45-3.61 |
| TG+ GG | 56.25 | 62.3 |  | 0.77 | 0.27-2.19 |
| Rec |  |  |  |  |  |
| TT+TG | 56.25 | 86.8 | <0.01 | 0.19 | 0.06-0.57 |
| GG | 43.75 | 13.2 |  | 5.12 | 1.73-15.16 |
| rs111521887/*TOLLIP* | | | | | |
| Dom |  |  |  |  |  |
| CC | 12.5 | 11.5 | 1 | 1.09 | 0.22-5.21 |
| CG+GG | 87.5 | 85.5 |  | 0.91 | 0.19-4.34 |
| Rec |  |  |  |  |  |
| CC+CG | 100 | 100 | NA | NA | NA |
| GG | 0 | 0 | NA | NA | NA |

Genotype frequencies were compared by Fisher exact test (p). Data presented with percentage Significant differences were demonstrated when p <0.05. Single Nucleotide Polymorphism (SNP); Patients with Combined Pulmonary Fibrosis and Emphysema Syndrome (CPFE); Pulmonary Aging Cohort subjects (PAC); Dominant model (Dom); Recessive model (Rec); Odds ratio (OR); Confidence interval 95 % (CI 95%); Not apply (NA).

**ST 6. Association between clinical variables of IPF and genotypes (TT+TG *vs.* GG) of *FAM13A* rs260955**

|  | Estimated Regression Coeficcient | p | CI 95% | |
| --- | --- | --- | --- | --- |
| Univariate | | | | |
| Macrophages (%) | -0.03 | 0.34 | -0.03 | 0.12 |
| Lymphocytes (%) | -0.02 | 0.55 | -0.1 | 0.04 |
| Atypical tomographic pattern | 0.64 | 0.32 | -0.68 | 1.89 |
| FVC (%) | 0.001 | 0.94 | -0.03 | 0.03 |
| DLco (%) | 0.01 | 0.44 | -0.01 | 0.04 |
| Multivariate | | | | |
| Macrophages (%) | -0.07 | 0.47 | -0.27 | 0.14 |
| Lymphocytes (%) | -0.06 | 0.38 | -0.22 | 0.08 |
| Atypical tomographic pattern | 1.73 | 0.97 | -0.08 | 3.85 |
| FVC (%) | 0.01 | 0.59 | -0.04 | 0.08 |
| DLco(%) | 0.02 | 0.30 | -0.02 | 0.08 |

Univariate and multivariate logistic regression model between clinical variables associated with a worse prognosis in IPF and genotypes of *FAM13A* rs2609255 (TT+TG *vs.* GG), Forced Vital Capacity (FVC), DLco (pulmonary diffusion capacity). p value <0.05.
